# Conditional Changes in Brood Size and Speed of Development After Long-term Laboratory Culturing of *C. elegans* Wild-type Strains

**DOI:** 10.17912/micropub.biology.001693

**Published:** 2025-10-21

**Authors:** Jocelyn M. Mertz, Ashley Kim, Bruce Wightman

**Affiliations:** 1 Muhlenberg College, Allentown, Pennsylvania, United States

## Abstract

Prolonged culturing of laboratory animals allows for genetic changes to occur in a population, potentially leading to phenotypic variation in designated wild-type strains. We discovered that two of our laboratory's *C. elegans* wild-type strains have undergone significant alterations in brood size and speed of development. One wild-type-derived strain displays a conditional increase in brood size, while a second strain exhibits a conditional decrease in brood size. The decrease in brood size in one strain is accounted for by a similar decrease in spermatogenesis. We also found that our two derived laboratory strains differ in speed of development. The strain with increased brood size displayed a faster progression during larval development, while the strain with decreased brood size displayed a slower progression of development. Therefore, both adaptive and non-adaptive changes in wild-type strains may occur after prolonged culturing.

**Figure 1. Brood Sizes and Developmental Progression in Wild-type Strains:**
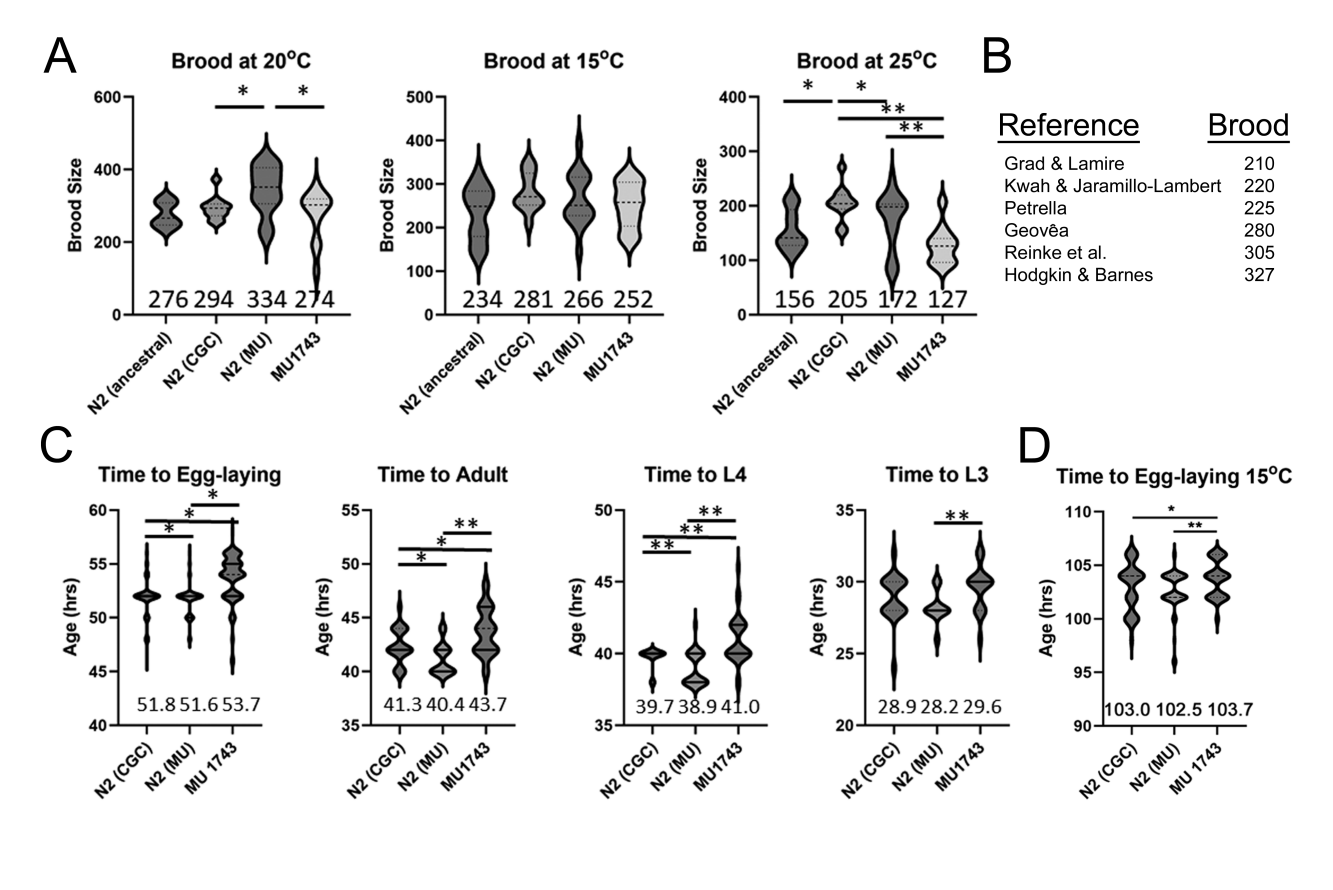
(A) Panels display hermaphrodite brood sizes for wild-type strains described in the text grown at 20 ^o^ C, 25 ^o^ C, and 15 ^o^ C. The mean brood size is displayed numerically for each strain at the bottom of each plot. Violin plots represent data from 10-17 independent trials for each strain and condition, with horizontal lines demarcating median and quartile values. (B) Wild-type brood size at 20 ^o^ C reported in six unrelated published studies. (C, D) Time of development plots showing ages in hours after continuous growth at 25 ^o^ C (C) or 15 ^o^ C (D) of synchronized populations. Violin plots represent data from 21-34 animals for adult, L4 and L3 milestones, and from 65-151 animals for egg-laying maturity, for each strain and condition. Egg-laying milestone was the accumulation of eggs in the uterus; Adult milestone was formation of an everted, mature vulva; L4 milestone was a fully inverted “Christmas-tree” shape vulva; L3 milestone was expansion of the ventral arms of the proliferating gonad. Ages in all assays are hours after egg-lay. We scored populations every two hours over the relevant time-period for each milestone. We evaluated statistical significance by unpaired t-test with Welch's correction: * p < .05; ** p < .01.

## Description

Laboratory animals under domestication are expected to evolve over time, particularly high-fertility r-selected species like *Caenorhabditis elegans* and *Drosophila melanogaster* (Sterken et al., 2015) *.* Some genetic changes may reflect unintentional selective events, when specific variants of the original strain are chosen for desirable culturing properties. For example, the *C. elegans* wild-type reference strain, N2 , carries a derived allele for the *npr-1* neuropeptide receptor that suppresses normal bordering behavior on bacterial lawns (Reddy et al., 2009; Weber et al., 2010; Zhao et al., 2015). Pleiotropies associated with selected variants may contribute to permanent alterations in the phenotypic properties of the strain. Indeed, genome sequencing of wild-type *C. elegans* variants separated in culture for about 40 years revealed over 1200 mutations, accompanied by phenotypic changes in time of embryogenesis, rate of egg production, and feeding behavior (Weber et al., 2010). It is unclear how many of these changes reflect selective events or neutral or deleterious consequences of genetic drift (García-Dorado et al., 2007). In principle, continued culturing in nutrient-rich conditions should select for improved fecundity--differential reproductive success should favor allele combinations that increase proliferation.

In the course of performing controls for an unrelated study, we discovered that our laboratory wild-type N2 strain, hereafter MU1743 , displayed a lower brood size than previous wild-type controls. The presence of a frozen isolate of N2 in our laboratory collection, designated N2 (MU), allowed us to compare MU1743 to the earlier wild-type strain from which it derived. MU1743 had an average brood size of 274 at 20 ^o^ C, considerably lower than the N2 (MU) average brood of 334 at 20 ^o^ C (Fig 1A). The brood size of MU1743 was similar to that of N2 (ancestral), a frozen isolate of the 1968 strain established by Sydney Brenner (mean 276, Fig. 1A ; reported to be 260 on WormBase). Freshly-ordered N2 worms from the *Caenorhabditis* Genetics Center, designated N2 (CGC), had an average brood size of 294 ( Fig. 1A ), somewhat lower than the advertised brood size of 350 ( *Caenorhabditis* Genetics Center), but similar to that reported in other studies ( Fig. 1B ). This analysis also demonstrated that the N2 (MU) strain had a significantly higher brood size than the N2 (CGC) wild-type control. At 25 ^o^ C, the reduced brood size for MU1743 was more evident: MU1743 average of 127 (s.d.=33), compared to N2 (CGC) and N2 (MU) averages of 205 (s.d.=30) and 172 (s.d.=50), respectively ( Fig. 1A ). No brood size difference was observed when animals were grown at 15 ^o^ C, indicating that the brood-size difference is environmentally-conditional.

Strain MU1743 diverged from N2 (MU), the lab's frozen wild-type isolate, approximately ten years ago. Since that time, it was continuously cultured under standard laboratory conditions, undergoing hundreds of generations with several periods of prolonged starvation. The history of N2 (MU) is less certain, but it also derived from an N2 isolate at the *Caenorhabditis* Genetics Center. Given the changes in brood size in comparison to different wild-type isolates performed in parallel under identical conditions, we conclude that the brood size differences reflect genetic changes in the strains. Therefore, prolonged culture produced two divergent outcomes: increased fecundity in N2 (MU) and decreased fecundity in MU1743 .

Our observations prompted us to review the brood sizes for N2 wild-type *C. elegans* reported from various laboratories ( Fig. 1B ). Among six arbitrarily-chosen studies, brood sizes for N2 at 20 ^o^ C ranged from 210 to 327, considerably below the presumptive 350 brood size claimed for the N2 strain. Among these studies, the average reported N2 brood size at 20 ^o^ C was 261 (s.d.=45.1), similar to our values for N2 (ancestral) and MU1743 . Although we do not have access to all the original data from these studies, the magnitude of these differences suggests statistical significance. These brood size differences among different laboratories have several possible explanations: environmental conditions of culture such as humidity or plate age, bacterial food quality or density, mechanical handling of parent, differences in trial censoring, or accumulated genetic changes in a particular laboratory isolate.

To explain the reduced brood size of MU1743 , we considered the possibility of reduced sperm production (L'Hernault, 2006). MU1743 produced unfertilized oocytes at the end of its reproductive life, just as the standard N2 strain does, suggesting an inability to fertilize eggs. We counted the number of sperm nuclei in individual spermatheca by DAPI staining in both N2 (MU) and MU1743 hermaphrodites that were grown at 20 ^o^ C. We found that N2 (MU) produced an average of 154.4 sperm per spermathecum (s.d.=14.1; consistent with an expected brood of 309), while MU1743 produced an average of 111.2 sperm per spermathecum (s.d.=34.7; consistent with a brood of 222; p<<.001). Therefore, a decline in sperm production appears to account for the reduced brood size in the diverged MU1743 , similar to spermatogenesis defects reported in other animals (Weeks et al., 2009).

Brood reduction could be coupled to changes in the rate of developmental progression. Accelerated maturation may offset lower brood size by allowing earlier reproduction (Aprison and Ruvinsky, 2016). To evaluate the speed of developmental progression in our strains, we determined the age at which animals begin producing eggs, as well as when they reach adult, L4, and L3 milestones at 25 ^o^ C ( Fig. 1C ). We found that MU1743 was slower than N2 (CGC) controls to begin laying eggs (114 minute delay), while N2 (MU) was slightly faster (12 minutes earlier). In order to determine whether this was the result of an earlier change in the speed of development, we measured the age at which strains reached earlier milestones. Time to adulthood was 144 minutes slower for MU1743 and 54 minutes faster for N2 (MU). Similarly, MU1743 reached an L4 milestone 78 minutes slower and N2 (MU) reached the same L4 milestone 48 minutes faster. In contrast, there was no significant difference between N2 (CGC) controls and the two laboratory-derived strains for an L3 milestone (small differences that appear in Fig. 1C are not significant; p=0.12 and p=0.17 for N2 (CGC) comparisons to N2 (MU) and MU1743 , respectively, by unpaired t test with Welch's correction). We also noted that about 10% of synchronized MU1743 populations consisted of larval laggards that were one to two stages behind the population. These animals were censored from our analysis and were not explored further, but this observation suggests a more significant low-penetrance developmental compromise in the slower, lower-brood MU1743 strain. Taken together, these data demonstrate a progressive late-larval developmental delay in MU1743 , and a progressive late-larval acceleration in N2 (MU).

Because the brood-size defect was temperature-sensitive, we explored whether the delayed and accelerated development phenotypes were similarly conditional. When grown at 15 ^o^ C, N2 (MU) was not significantly different from N2 (CGC), but MU1743 still showed a 43 minute developmental delay to reach egg-laying maturity ( Fig. 1D ). Therefore, the increased developmental speed of N2 (MU) is conditional, but the developmental delay of MU1743 is not, potentially uncoupling the brood size effect from the developmental delay phenotype.

In summary, prolonged laboratory culture drove opposite trajectories in two N2 isolates. N2 (MU) acquired both a higher brood and faster development, while MU1743 acquired both a lower brood and slower development. The former is expected as the result of selection over time for improved reproductive success, but the latter suggests a different genetic history. Founder effect and genetic drift during culturing may allow for the fixation of deleterious alleles in a self-fertile organism, similar to the phenomenon of inbreeding depression (Charlesworth and Willis, 2009; Santos et al., 2012). These results demonstrate that “wild-type” strains accumulate substantial divergence, even within a single laboratory, and that such changes can confound quantitative phenotypes. Our observations underscore the best practices of reconstituting wild-type controls from isogenic strains whenever possible, the periodic re-establishment of wild-type strains in laboratories, and caution when comparing results across laboratories.

## Methods

*C. elegans* strains were cultured on NGM agar and fed OP50 bacteria (Stiernagle, 2006). Temperature was maintained in Fisher Scientific refrigerated incubators, monitored with precision mercury thermometers in addition to the integral electronic temperature probes.

Brood size assays were conducted by picking individual L4 hermaphrodites to individual plates, passaging them to fresh plates for three to four days. Next generation progeny were counted once they had reached L4 and adults and totaled for each trial animal.

Sperm counts were determined by DAPI staining and fluorescence microscopy using a Nikon UD microscope. For each fixed specimen, individual spermatheca were photographed in a series of four to six Z-plane sections, with individual sperm nuclei counted by hand and totaled for each specimen.

Developmental progression studies were performed by picking 8-10 gravid hermaphrodites, grown at the desired temperature, to individual NGM plates spread with OP50 bacteria and allowing them to lay eggs for two hours. No difference in time of hatching was observed among the three strains evaluated. The synchronized cultures were incubated at the desired temperature and examined every two hours for specific anatomical landmarks indicative of particular stages (detailed in legend for Fig. 1). For adult, L4, and L3 milestones, we examined anatomy via DIC microscopy using a Nikon UD microscope. We scored egg-laying maturity using a Leica dissecting microscope.

Statistical tests were performed using Microsoft Excel and GraphPad Prism software; plots were produced by Prism.

## References

[R1] Aprison EZ, Ruvinsky I (2016). Sexually Antagonistic Male Signals Manipulate Germline and Soma of C.&nbsp;elegans Hermaphrodites.. Curr Biol.

[R2] Charlesworth D, Willis JH (2009). The genetics of inbreeding depression.. Nat Rev Genet.

[R3] García-Dorado A, Avila V, Sánchez-Molano E, Manrique A, López-Fanjul C (2007). The build up of mutation-selection- drift balance in laboratory Drosophila populations.. Evolution.

[R4] Gouvêa DY, Aprison EZ, Ruvinsky I (2015). Experience Modulates the Reproductive Response to Heat Stress in C. elegans via Multiple Physiological Processes.. PLoS One.

[R5] Grad LI, Lemire BD (2003). Mitochondrial complex I mutations in Caenorhabditis elegans produce cytochrome c oxidase deficiency, oxidative stress and vitamin-responsive lactic acidosis.. Hum Mol Genet.

[R6] Hodgkin J, Barnes TM (1991). More is not better: brood size and population growth in a self-fertilizing nematode.. Proc Biol Sci.

[R7] Kwah Ji Kent, Jaramillo-Lambert Aimee (2023). Measuring Embryonic Viability and Brood Size in <em>Caenorhabditis elegans</em>. Journal of Visualized Experiments.

[R8] L'Hernault S.W. (2006). Spermatogenesis. WormBook.

[R9] Petrella LN (2014). Natural variants of C. elegans demonstrate defects in both sperm function and oogenesis at elevated temperatures.. PLoS One.

[R10] Reddy KC, Andersen EC, Kruglyak L, Kim DH (2009). A polymorphism in npr-1 is a behavioral determinant of pathogen susceptibility in C. elegans.. Science.

[R11] Reinke SN, Hu X, Sykes BD, Lemire BD (2010). Caenorhabditis elegans diet significantly affects metabolic profile, mitochondrial DNA levels, lifespan and brood size.. Mol Genet Metab.

[R12] Santos J, Pascual M, Simões P, Fragata I, Lima M, Kellen B, Santos M, Marques A, Rose MR, Matos M (2012). From nature to the laboratory: the impact of founder effects on adaptation.. J Evol Biol.

[R13] Sterken MG, Snoek LB, Kammenga JE, Andersen EC (2015). The laboratory domestication of Caenorhabditis elegans.. Trends Genet.

[R14] Stiernagle Theresa (2006). Maintenance of C. elegans. WormBook.

[R15] Weber KP, De S, Kozarewa I, Turner DJ, Babu MM, de Bono M (2010). Whole genome sequencing highlights genetic changes associated with laboratory domestication of C. elegans.. PLoS One.

[R16] Weeks SC, Reed S, Ott D, Scanabissi, F. Inbreeding effects on sperm production in clam shrimp (Eulimnadia texana). Evolutionary Ecology Research, 2009, 11: 125–134.

[R17] Zhao Y, Long L, Xu W, Campbell RF, Large EE, Greene JS, McGrath PT (2018). Changes to social feeding behaviors are not sufficient for fitness gains of the Caenorhabditis elegans N2 reference strain.. Elife.

